# Differences in soil biological activity by terrain types at the sub-field scale in central Iowa US

**DOI:** 10.1371/journal.pone.0180596

**Published:** 2017-07-07

**Authors:** Amy L. Kaleita, Linda R. Schott, Sarah K. Hargreaves, Kirsten S. Hofmockel

**Affiliations:** 1 Agricultural & Biosystems Engineering, Iowa State University, Ames, Iowa, United States of America; 2 Biological Systems Engineering, University of Nebraska, Lincoln, Nebraska, United States of America; 3 Ecological Farmers Association of Ontario, Guelph, Ontario, Canada; 4 Formerly: Ecology, Evolutionary and Organismal Biology, Iowa State University, Ames, Iowa, United States of America; 5 Environmental Molecular Sciences Laboratory, Pacific Northwest National Laboratory, Richland, Washington, United States of America; Tennessee State University, UNITED STATES

## Abstract

Soil microbial communities are structured by biogeochemical processes that occur at many different spatial scales, which makes soil sampling difficult. Because soil microbial communities are important in nutrient cycling and soil fertility, it is important to understand how microbial communities function within the heterogeneous soil landscape. In this study, a self-organizing map was used to determine whether landscape data can be used to characterize the distribution of microbial biomass and activity in order to provide an improved understanding of soil microbial community function. Points within a row crop field in south-central Iowa were clustered via a self-organizing map using six landscape properties into three separate landscape clusters. Twelve sampling locations per cluster were chosen for a total of 36 locations. After the soil samples were collected, the samples were then analysed for various metabolic indicators, such as nitrogen and carbon mineralization, extractable organic carbon, microbial biomass, etc. It was found that sampling locations located in the potholes and toe slope positions had significantly greater microbial biomass nitrogen and carbon, total carbon, total nitrogen and extractable organic carbon than the other two landscape position clusters, while locations located on the upslope did not differ significantly from the other landscape clusters. However, factors such as nitrate, ammonia, and nitrogen and carbon mineralization did not differ significantly across the landscape. Overall, this research demonstrates the effectiveness of a terrain-based clustering method for guiding soil sampling of microbial communities.

## Introduction

Microbial biomass and activity are both critical to, and sensitive indicators of, ecosystem health [1]. In production agricultural systems, microbial functions are especially important due to the high nutrient demands of plants.

Precision agriculture is a domain which has focused on techniques for site-specific management of nutrients, water, weeds, and disease, largely without much attention to the microbial communities that drive many of the plant and soil processes critical to agricultural productivity and agricultural impacts on the environment. Welbaum et al. [2] point out the need for building on the advancements of precision agriculture and integrated pest management by including an emphasis in microbial ecosystem management in production agriculture. The authors call for improved methods of microbial monitoring for this purpose. This is echoed by Shennan [3] who points out the need for considering ecological interactions at a range of scales to inform and optimize agroecosystem management.

Soil microorganisms exhibit high spatial variability, even in uniformly managed agroecosystems [4], making it difficult to capture their abundance and function through standard random plot sampling. Samples are limited to specific points in time and space, which may not be representative or informative. The spatial patterns exhibited can vary significantly depending upon the spatial resolution of the sampling itself (eg. [5–7]). The influence of environmental properties may also be a function of scale [8], and may change over time as ambient conditions, particularly soil water content, change (e.g. [9–10]). In fact, shifts in microbial biomass can occur slowly in response to soil drying, but rapidly–within hours or days–in response to a rainfall event [11]. Hence, capturing microbial responses through field-scale soil sampling is not a trivial task. Ideally, observations would be made frequently (Landesman and Dighton [11] suggest hourly) and with a high spatial density (micrometre—meter scales [12]). However, this is rarely feasible given inevitable limitations of time and/or money. Thus, understanding how patterns in more readily observable properties relate to soil microbial properties may help to guide sampling design.

Topography is known to be a significant control on microbial communities, due primarily to the influence of topography on soil moisture and organic carbon [9, 13, 14]. For example, Florinsky et. al [9] measured the strength of association between individual topographic indices (slope, aspect, curvature, flow contributing area, topographic index, and stream power index) and soil microbial properties (microbial biomass, most probable number, denitrifier enzyme activity, denitrification rate, and microbial respiration rate). By using these relationships in a regression, they were able to develop predictive maps of microbial biomass and denitrifier enzyme activity. The strength of topographic controls varied by soil wetness conditions, decreasing to statistically insignificant relationships between topography and microbial properties under dry conditions.

The use of soil physicochemical characteristics to inform soil microbial sampling or activity mapping have produced differing results. Research has shown that in agricultural soils, microbial biomass and respiration are directly related to spatial patterns in soil pH, dissolved organic carbon, and total organic carbon [4]. In contrast, Katsalirou et al. [15] found weak spatial structure of organic carbon in cultivated soil, and strong positive linear relationships between soil organic carbon and microbial properties in uncultivated soils. Together these studies demonstrate the limitations of traditional geostatistics for soil sampling designs, and the need to use alternative parameters to guide sampling designs. For example, Cavigelli et al. [16] attempted to address how soil microbial property sampling schemes could be guided by soils information: drainage class, soil series, soil map unit, and texture of the Ap horizon. A relatively small proportion of measured soil microbial properties showed soil type effects, suggesting that terrain attributes and other factors that impact soil moisture dynamics may be most important [16]. Because of the high degree of microbial functional diversity and the strong influence of environmental factors, conventional soil classification may not provide a good indicator of microbial activity [17]. Instead, soil structure, which is intimately related to soil moisture and oxygen availability, may be better predictors of microbial biomass and function [12].

Peigné et al. [18] suggested that sampling design should be based upon historical exhaustive sampling of the soil and/or microbial activity to establish their spatial structure and to delineate “zones” of expected high, medium, and low values of the parameter(s) of interest. While useful, the pre-sampling required to design a sampling scheme in this way may be time- and cost- prohibitive in many cases. Van Arkel and Kaleita [19] illustrated that soil moisture sampling could be guided by a clustering analysis on soil and terrain attributes. Given the importance of soil moisture on microbial activity, and the body of literature suggesting that microbial patterns are related to a combination of soil factors and water-related terrain factors, it is reasonable to hypothesize that microbial sampling could be effectively guided with the same or similar approach. A number of studies have investigated “landform segmentation” for delineating zones related to soil microbial activity (enumerated in Pennock [14]), however, these have generally focused only on topographical attributes, and have not emphasized development of a practical methodology for sampling. Tajik et al. [20] considered both soil and terrain variables to predict soil enzyme activity, but the soil variables were derived from extensive soil sampling, which diminishes the practicality of this approach.

In this study, we use the Van Arkel and Kaleita [19] approach to classifying a landscape into moisture-related groups on the basis of readily available terrain and soil attributes, and examine if there is a detectable difference in microbial communities that reside in the different landscape classes within a 60-ha corn/soybean field in central Iowa, US. We use a self-organizing map to determine the number of landscape classes evident in the study area, followed by K-means clustering to partition the landscape into that number of classes. We then analyse soil samples taken from within those classes to deterring if there are statistically significant differences in microbial biomass and activity. If so, this would suggest a grouping or clustering algorithm could be useful for guiding soil microbial sampling design.

## Methods

### Site information

The study site is a roughly 77-ha conventionally farmed field in Story County, central Iowa, with a crop rotation of corn and soybeans. The elevation in the field varies by approximately 6 m. While the elevation changes are moderate, the site includes different landscape positions, including ridges, toeslopes, and closed depressions. According to the National Cooperative Soil Survey (NCSS), there are six main soil types in this field: two loams, three clay loams, and one silty clay loam, located in the closed depressions. However, NCSS soil type delineations are not precise at this fine resolution (e.g. [21]).

Elevation data for this field were obtained using a GPS receiver mounted on the all-terrain vehicle that pulled the EMI sled. Using Surfer® (Golden Software, Inc., Golden, Colorado), a 10 m grid of elevation data was interpolated from this elevation data. Slope, planar curvature, and slope aspect were then derived using Surfer®, which uses computational processed based on Moore et al. [22] to calculate terrain attributes. A 10 m grid was used based upon the finding by Yang [23] that this scale was adequate to describe field-scale soil moisture patterns at an adjacent field site; others have also suggested the 10 m scale is appropriate for managing spatially variable soil moisture [24].

In the absence of high-resolution soils data, we use electromagnetic inductance (EMI) as a proxy to identify changing soil properties. This noncontact sensor is sensitive to variations in several characteristics of the soil, including soil texture, soil moisture content, organic matter, and depth of clay pan. Consequently, the EMI data are not direct measures of any single soil property, but variations in EMI do reflect the heterogeneity in soil properties and for this reason are frequently used as a low-cost alternative to extensive soil sampling in applications where soil spatial variability is of interest [25]. Both horizontal (H-H) and vertical (V-V) conductances in units of milliSiemens/meter were gathered using an EMI sled pulled by an all-terrain vehicle. These data were collected in early spring when the field was saturated with snowmelt and spring rain, in order to minimize EMI differences due to soil moisture spatial variability. EMI data were interpolated with inverse distance weighting from a roughly 20 m average resolution (measurements were more dense in the direction of travel of the sled than in between sled passes) to the same 10 m landscape grid as the elevation data.

Thus, for each of the 7050 locations on the 10m grid across the study area, we had a six-element vector of terrain and soil characteristics that included: elevation, slope, slope aspect, planar curvature, H-H EMI, and V-V EMI. These were used as the inputs to the self-organizing map algorithm as described below. Summary statistics of these data for our site are given in Table 1.

**Table 1:** Terrain and soil characteristics across the study area.

*Attribute (units)*	*Range*	*Mean (Std. Dev)*
***Elevation (m)***	309.8–315.9	312.4 (1.3)
***Slope (%)***	0.0–4.6	1.2 (0.7)
***Slope aspect (degrees clockwise from north)***	0–360	178 (105)
***Plan curvature (m^-1^)***	-4.8–12.1	0.0 (0.2)
***H-H EMI***	14.9–90.7	47.0 (13.8)
***V-V EMI***	10.4–68.3	32.6 (11.3)

### Sampling location selection

A method of analysis gaining popularity in modeling natural processes is that of computational intelligence, specifically self-organizing feature maps (SOMs), a type of artificial neural network approach. SOM networks learn to arrange occurrences of similar input patterns from a high dimensional input space into a low dimensional lattice of ‘neurons’ in an output layer [26]. The end result is an output network layer (map) with contiguous neurons having similar patterns in their input data [27]. Mele and Crowley [28] successfully used self-organizing maps to demonstrate relationships among different soil biogeochemical properties that can be used to assess soil quality. An advantage of using SOMs to analyse ecological communities is that this network approach allows visualization of associations among data even when the associations occur in different dimensions of the data space [29]. In the case of spatial data, SOM allows for identification of relationships in the data regardless of the spatial proximity of sampling locations. Often, SOMs are combined with a clustering algorithm to find clusters of similarly behaving input data (e.g. [30]). Self-organizing maps may, thus, be useful as a way to identify groups of location in the field, which could be subsampled.

A thorough explanation of the SOM procedure can be found in Kohonen [26]. A set of *n* input vectors of *k* elements are presented to the SOM algorithm and the vectors then ‘self-organize’ into a two-dimensional map according to the following process. To begin the algorithm produces a random arrangement of the input vectors into a 2-dimensional lattice map. Neurons or nodes on the output map are represented by the vector ***m***_*i*_ = [*m*_*i*1_,*m*_*i*2_,…,*m*_*ik*_], (*i* = 1,2,…,*N*), where *N* is the number of neurons on the output map, *k* is the input vector dimension and *m*_*ik*_ represents the value of input data assumed (or later, computed through the updating process described below) for that node. The number of neurons on the map is user-specified and need not be the same as *n*.

The SOM is trained iteratively. At each iteration, the algorithm compares a randomly chosen input vector ***x*** to each node in the lattice, and the winning node, ***m***_*c*_, for that vector is chosen based on the formula

$$\|x_{input}-m_{c}\|={min}_{i}\{\left\|x_{input}-m_{i}\right\|\}.$$ (1)

Thus, the winning node is the node with the smallest Euclidean distance to the input vector. After finding the winning output node for each input vector, the node’s vector is updated according to the following equation:

$$m_{i}(t+1)=m_{i}(t)+h_{ci}(t)[x(t)-m_{i}(t)]$$ (2)

where *t* denotes the index of the iteration step, ***x****(t)* is the input sample of ***x***_*input*_ in the iteration *t*, and *h*_*ci*_*(t)* is called the neighborhood function around the winning node ***m***_*c*_. During training, *h*_*ci*_*(t)* is a decreasing function of the distance between the *i*^*th*^ and the *c*^*th*^ node. For convergence it is necessary that *h*_*ci*_*(t)* goes to 0 when *t* goes to infinity. The iteration then proceeds, and node vectors are updated at each iteration, with the end result that the final map has a distribution that represents the characteristics of all *n* of the *k*-dimensional input vectors in a two-dimensional space of *N* nodes.

To produce the input vectors from the elevation, EMI (H-H and V-V), slope, aspect, and curvature grid data, a matrix was built with each parameter in a different cloumn, and rows representing individual field locations in the 10 m grid, for a resulting 7050 x 6 matrix (*n* = 7050 and *k* = 6). In this way, a vector of terrain characteristics for each location was generated. This matrix was then fed to the SOM algorithm within the MATLAB SOM Toolbox 2.0 [31]. Different process options can be chosen within the SOM algorithm; in this study, the default parameters within the Toolbox were used. Because the magnitudes of the 6 columns of characteristic data varied, SOM development was based upon normalized data for each characteristic (this is an option within the Toolbox).

Using the SOM Toolbox, a unified distance matrix (U-matrix) was generated. The U-matrix shows the distance between the nodes and can be used to identify patterns within the data. Distance between nodes is an inverse representation of similarity; the larger the distance between two nodes, the more dissimilar are the characteristics of the grid points in those nodes. By observing the color difference in the map and using the color scale, one can see the difference in distances between nodes within the U-matrix. Colors corresponding to small numerical values show that the nodes are closely related, whereas colors corresponding to large numerical values show divisions within the input data. Based on this visualization, the number of different groups of data in the field characteristics can be inferred, because large separation suggest large differences in the data. As will be explained in more detail in section 3, three groups were assumed for this site.

A K-means clustering procedure was then used to separate both the SOM map neurons and the temporal and physical data matrices into different clusters containing points with similar characteristics. In the K-means algorithm, the initial ‘means’ of a k number of clusters is randomly selected from the input data set. Clusters are created by associating each input vector (grid point data) to the nearest mean. The nearest mean is determined by finding the smallest Euclidean distance between the input vector and the mean vector. The input vectors are then partitioned into clusters depending on their distances from the mean vectors. The geometric centre of each of the clusters becomes the new mean. This process continues until there is no change in cluster membership given additional iterations of the algorithm. Readers are referred to MacQueen [32] for further explanation of the K-means algorithm. The MATLAB SOM Toolbox contains a K-means clustering algorithm that was used for partitioning the neurons in the output layer of the SOM.

After applying the K-means clustering algorithm to both the SOM neuron data, the centroid vector of each cluster in each method was identified. As the name suggests, the centroids represent the centres (means) of the clusters created. Using the Euclidean distance formula, the input vector (corresponding to a single location) with the smallest distance from each centroid was identified. This input vector (location) was deemed the best matching unit (BMU) to the cluster centroid. These BMUs were then used as the critical sampling locations identified for this field. Final soil sampling locations thus included the BMU for each of the three groups, and an additional eleven sites within each group, distributed spatially across the field, for a total of 36 sites.

### Soil biological sampling and processing

At each of the 36 locations, we collected a single 7.6 cm (3 inch) soil core to a depth of 10 cm and stored it at 4°C until analysis. With permission from the land owner (Iowa State University), we collected soil samples on a single date, October 24, 2011, prior to the field being harvested. Prior to analysis, we homogenized soil samples to a particle size of at least 4mm and sub-sampled individual measurements. We measured gravimetric water content as soil mass loss upon drying at 60°C for 72 h, soil pH using 1:2 soil: water ratio, and total C and N by dry combustion (LECO TruSpec CN, St. Joseph, MI).

We also analysed each sample for soil microbial biomass, mineralization rates and available C and N pools. We measured microbial biomass C (MBC) and microbial biomass N (MBN) as the difference in K_2_SO_4_-extractable dissolved organic C (DOC) and total N (Shimadzu TOC-L CPH/CPN, Kyoto, Japan), respectively, between chloroform fumigated and non-fumigated subsamples using 15 g of soil extracted with 45 mL 0.5M K_2_SO_4_. We used the non-fumigated K_2_SO_4_-extractable DOC value to represent the soil available DOC concentration (modified from [33]). We used a calibration factor of 0.45 for MBC [34] and 0.54 for MBN [35].

We measured potential N mineralization as the difference between extractable inorganic N after a 28-day incubation (modified from [33]), and used non-fumigated samples to determine extractable inorganic pools of N at the beginning of the incubation. We measured extractable nitrate and ammonium concentrations in microplate format via spectrophotometry (BioTek Synergy HT plate reader, BioTek Instruments, Inc., Winooski, VT) [36]. We measured potential C mineralization by sampling CO_2_ concentrations in the headspace on days 1, 3, 7, and 10 using an infrared gas absorption analyser (LICOR) (modified from [33]). After each sampling, flasks were vented to prevent CO_2_ build-up and possible inhibition of aerobic respiration. C mineralization rates were, therefore, calculated as the concentration per day.

### Statistical analysis

We generated correlation statistics to compare, on a point-basis, the microbial indicators with the terrain attributes for the 36 sampled locations. On a cluster group basis, we used ANOVA to determine if the microbial data from each of the three groups are significantly different. Student’s t-tests were performed to determine if the BMU site provides a good estimate of the group mean.

## Results and discussion

Correlation analysis indicated that both terrain and soils attributes have similar trends to microbial indicators (Table 2). Overall, microbial indicators were most strongly correlated with the EMI data, with correlations ranging from 0.39 to 0.83 in magnitude. Specific carbon and nitrogen mineralization were negatively correlated with EMI, while the other indicators were positively correlated with EMI. Microbial indicators were also correlated, to a somewhat lesser degree, with elevation and slope. Plan curvature as an individual index was not correlated with any of the microbial indicators. Overall, specific N mineralization showed the least strong correlation with any of the landscape data, with no correlation coefficient greater than 0.39 in magnitude.

**Table 2:** Correlations between terrain and soil attributes and microbial indicators: extractable dissolved organic carbon (EOC; ug C g^-1^ soil), microbial biomass carbon (MBC; ug C g^-1^ soil), percent total soil carbon (%TC), microbial biomass N (MBN; ug N g^-1^ soil), percent total soil nitrogen (%TN), specific Carbon mineralization (Cmin), and specific Nitrogen mineralization (Nmin).

	*EOC*	*MBC*	*%TC*	*MBN*	*%TN*	*Cmin*	*Nmin*
***Elevation***	-0.49	-0.64	-0.68	-0.63	-0.63	0.38	0.30
***Slope***	-0.39	-0.63	-0.68	-0.60	-0.69	0.57	0.28
***Slope aspect***	-0.17	0.04	-0.07	0.01	0.02	-0.24	-0.24
***Plan curvature***	0.12	0.04	0.06	0.06	0.04	-0.10	0.04
***H-H EMI***	0.42	0.81	0.74	0.75	0.79	-0.48	-0.39
***V-V EMI***	0.45	0.83	0.79	0.79	0.82	-0.53	-0.44

**Fig 1:**
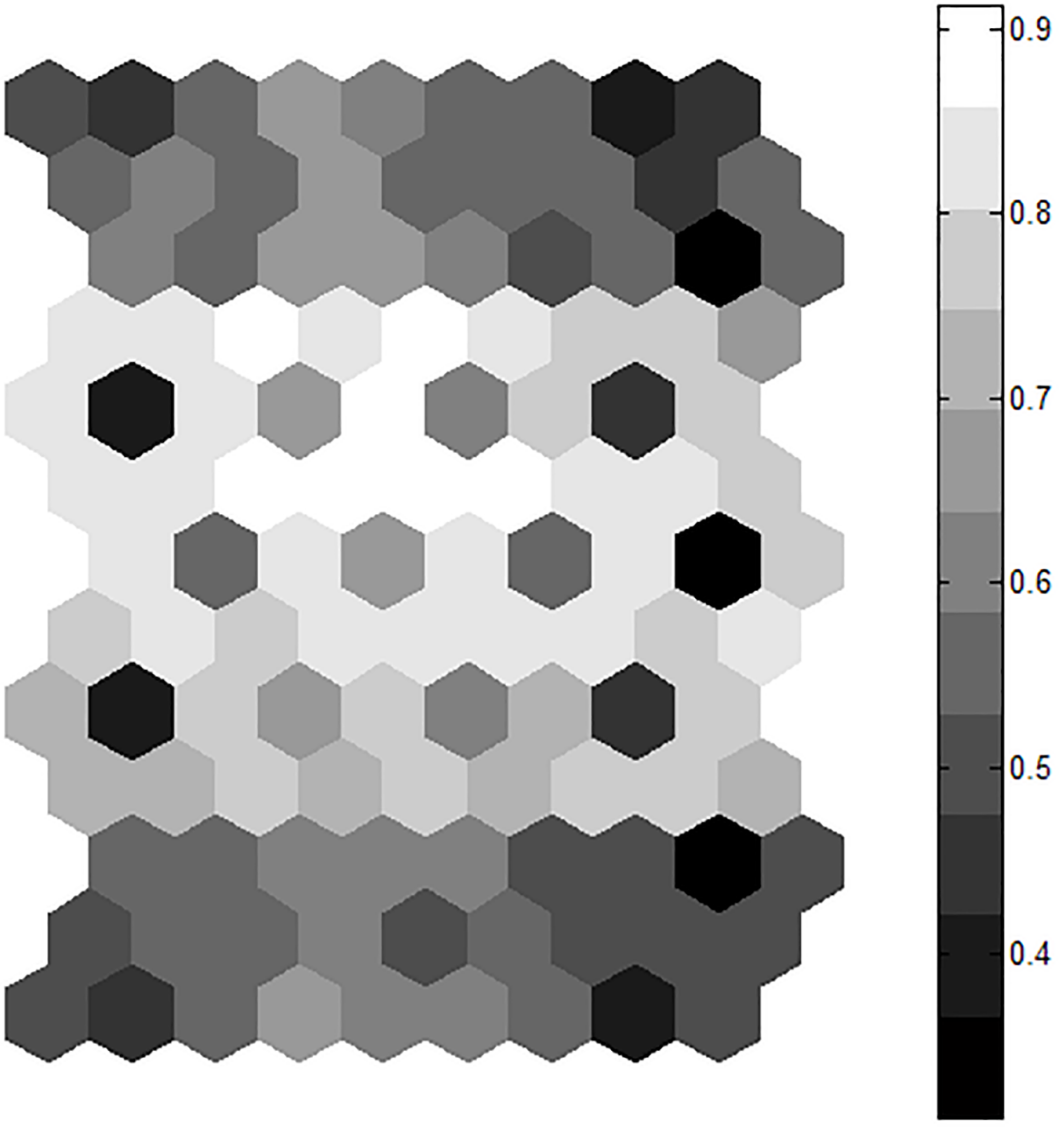
U-matrix for the terrain and EMI data for the study site. The legend bar to the right of the U-matrix denotes the Euclidean distance in input data between neighbouring nodes. Lower values indicate more similarity with neighbouring nodes.

The SOM U-matrix was analysed to determine the appropriate number of groups or clusters in the field, based on the terrain and EMI data. Fig 1 shows the U-matrix. In this case, strong division between top and bottom sections of the U-Matrix suggests two groups. However, the middle of the U-matrix is wide, with some closely related nodes, and may suggest a third group. We thus assumed there were three groups in this field, and used the K-means clustering on the self-organizing map to segment the field into three groups accordingly.

**Fig 2:**
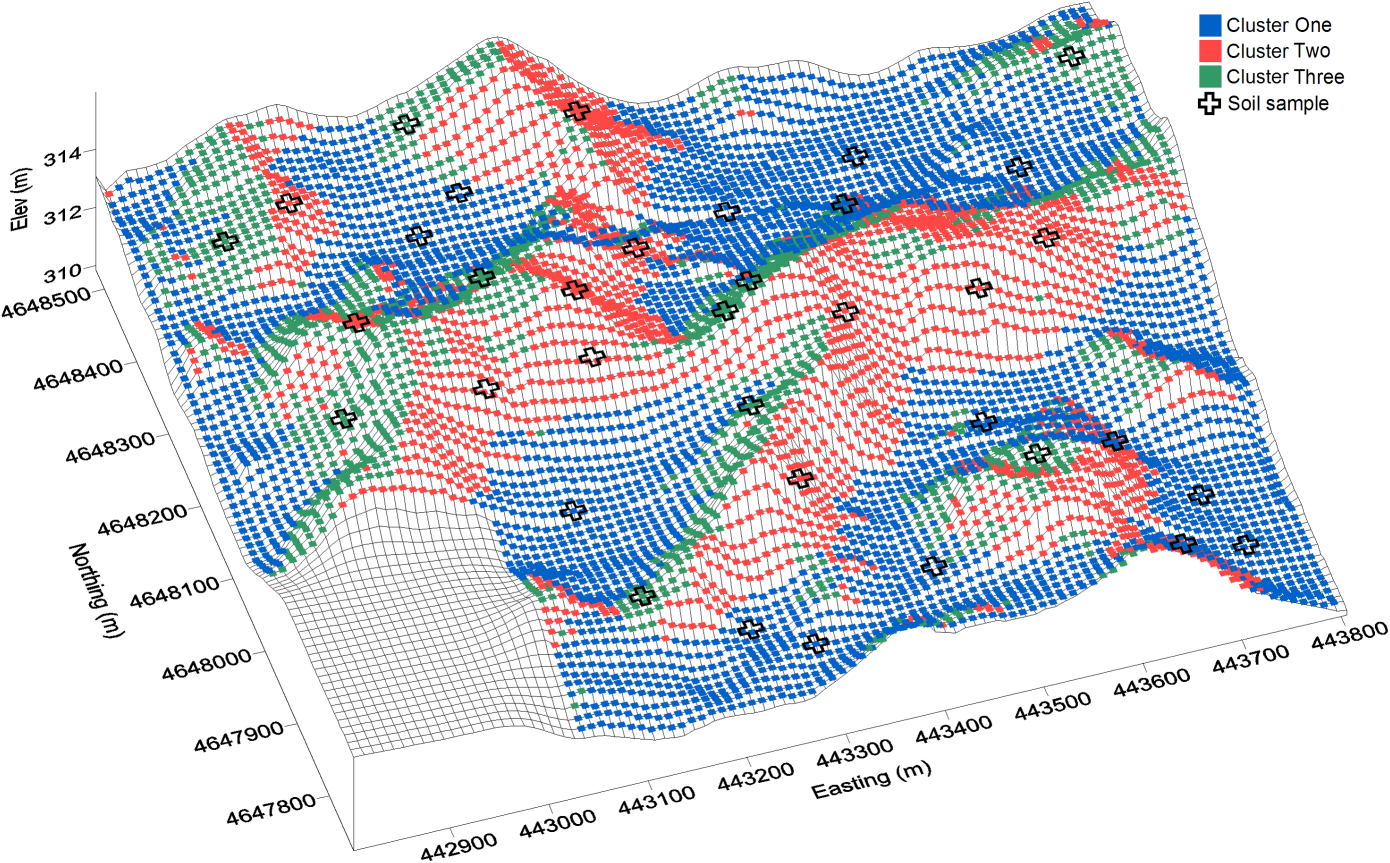
Distribution of clusters of terrain and soils data in the field. Soil sampling locations are marked. Each colored dot represents the centre point on a 10 meter grid.

**Fig 3:**
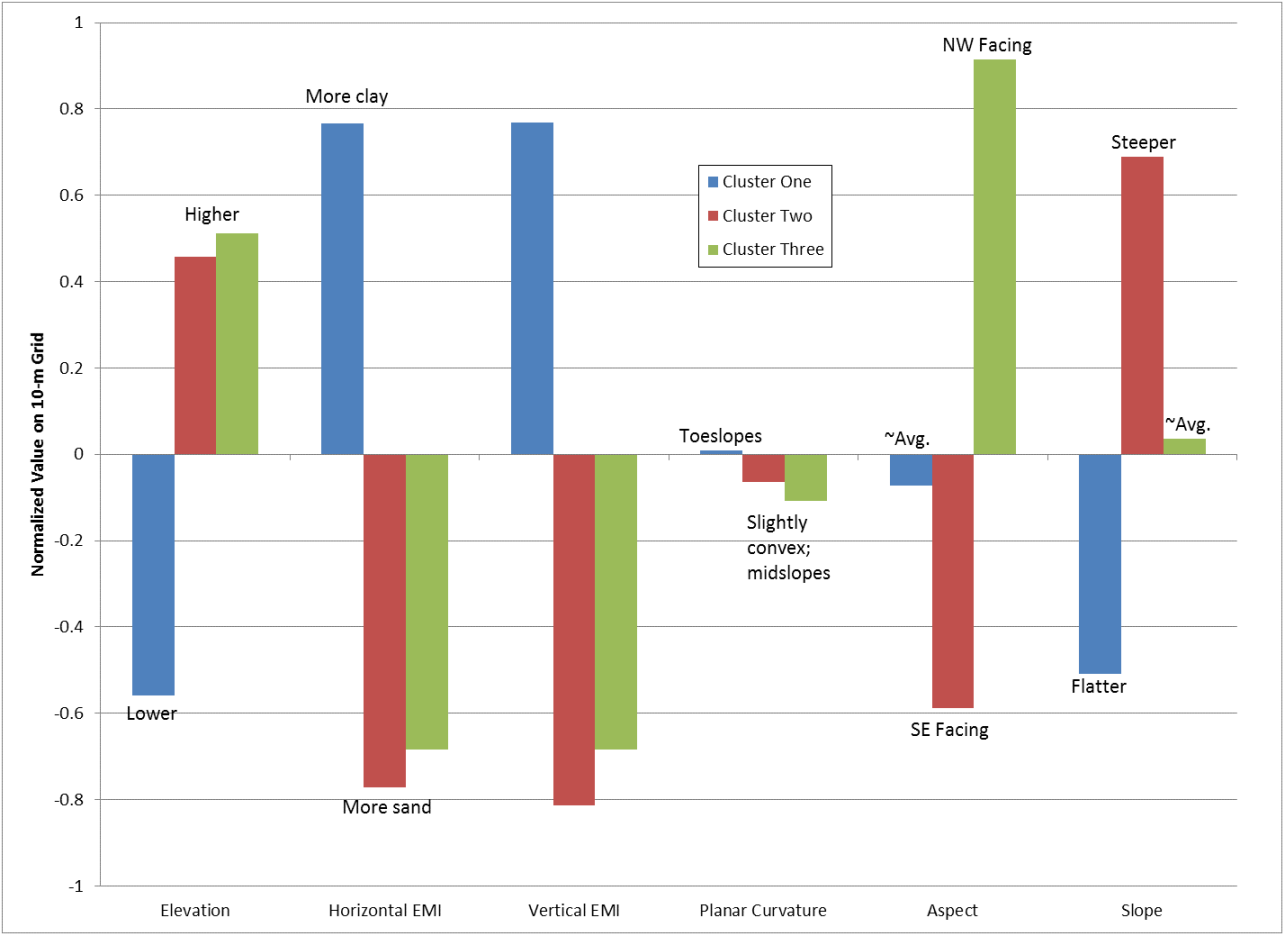
Average characteristics of the terrain and EMI data for each cluster and their interpretation. For development of the SOM, each attribute for all locations on the 10-meter grid was normalized to a mean of zero and standard deviation of one; the normalized values are shown here.

The characteristics and spatial distribution of these groups were then reviewed. Fig 2 shows the spatial distribution in the field of the three groups resulting from the K-means clustering, and Fig 3 shows the characteristics of the three groups. Cluster one is primarily comprised of the potholes and toe slopes with higher EMI soils, which most likely have higher clay content. Cluster two and three are at higher elevations and have lower EMI values. These two groups are quite similar except that they face different directions; cluster two consists mainly of the southern facing slopes, whereas cluster three includes the northeast facing slopes. Cluster two has steeper slopes while slopes in cluster three are close to the field average.

**Fig 4:**
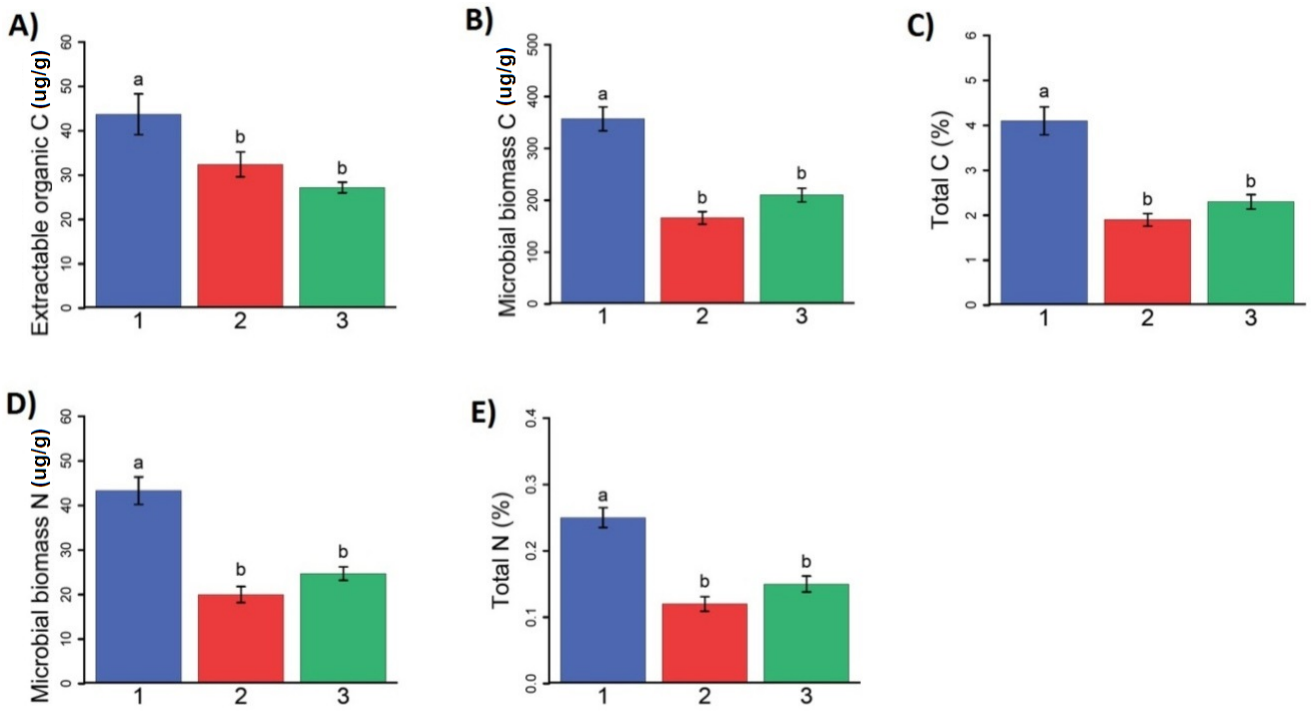
**Mean cluster values for (A) extractable dissolved organic carbon (ug C g**^**-1**^ **soil), (B) microbial biomass carbon (ug C g**^**-1**^ **soil), (C) percent total soil carbon, (D) microbial biomass N (ug N g**^**-1**^ **soil), and (E) percent total soil nitrogen.** A significance level of p<0.05 was used for evaluation. Colors indicate location of soil clusters, where blue is cluster 1, red is cluster 2 and green is cluster 3.

The soil analysis results were then analysed by cluster. Fig 4 shows mean cluster values of microbial analysis. When grouped by cluster, mean values of DOC, MBC, MBN, TC, TN were significantly greater in cluster one than in clusters two and three (p<0.05). Mean DOC for cluster one was more than 1.3 times larger than either of the other two clusters. Mean MBC for cluster 1 was 1.6 times greater than for clusters 2 and 3, and mean MBN was 1.75 greater. Finally for TN and TC, the mean for cluster 1 is at least 1.8 and 1.6 times greater, respectively, than the other two clusters.

**Fig 5:**
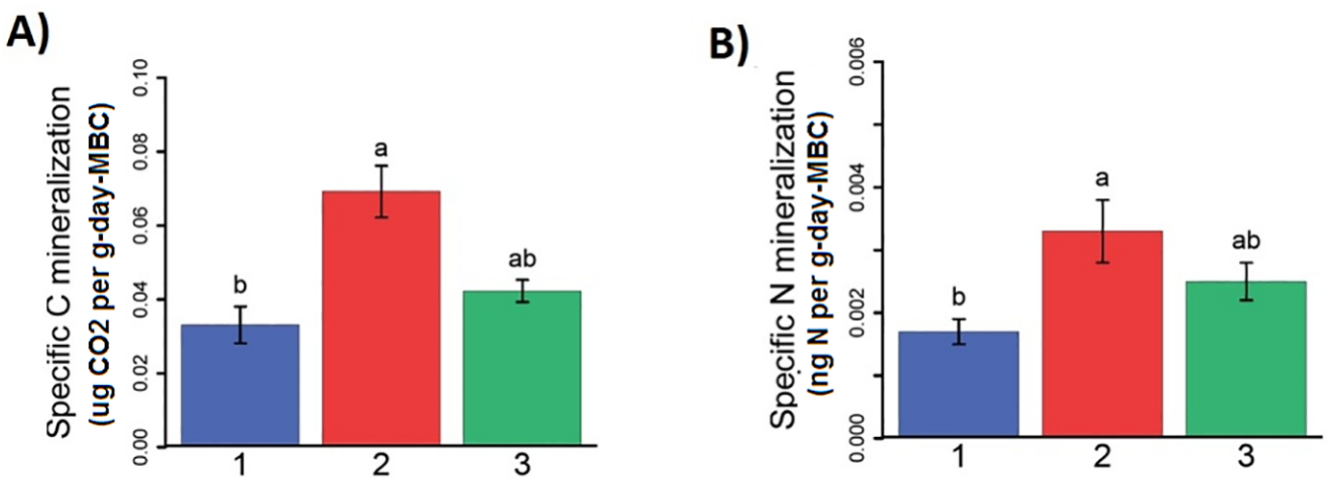
**Mean cluster values for (A) specific carbon mineralization and (B) specific N mineralization.** A significance level of p<0.05 was used for evaluation. Colors indicate location of soil clusters, where blue is cluster 1, red is cluster 2 and green is cluster 3.

Potential rates of C and N mineralization, ammonia and nitrate concentrations, and the ratio of MBC to MBN did not differ among the three clusters. However, when the C and N mineralization rates were computed on a per unit MBC basis, the mass specific rates of C and N mineralization were significantly different (p< 4.0e-5 and p< 0.051, respectively) between Clusters 1 and 2. Cluster 1 had the lowest rates and cluster 2 had the highest. Cluster 3 was statistically similar to both Cluster 1 and Cluster 2. These are shown in Fig 5.

With respect to microbial activity indicators, these results indicate that only two clusters are evident at this site. This suggests that two strong clusters in the SOM would have been sufficient; the only difference between clusters 2 and 3 in the SOM input data is the primary direction the hillside faces, and cluster 2 was steeper than cluster 3. The microbial activity within these two clusters was for the most part the same.

The clustering of sample locations based on small-scale topographic and soil features effectively identified differences in microbial abundance and function between two groups (cluster 1 versus clusters 2 and 3 combined). Potholes and toe slopes (cluster 1) had greater organic C, but these areas also had lower specific N mineralization and specific microbial respiration rates compared to the upslope areas (clusters 2 and 3). This indicates that under the wetter conditions of cluster one there is probably leaching of soluble C that is readily available for microbial assimilation and use for microbial growth, as suggested by the greater EOC and MBC in the pothole soils. Corre et al. [37] had similar results and attributed the differences in microbial biomass between the upper and lower slopes to the availability of materials for mineralization. The lower slopes have more redistributed soil material due to surface water flow, so these areas are able so support a higher microbial biomass. It is likely that the wet, nutrient-rich conditions in the potholes allow microbial substrates to diffuse throughout the soil, supporting a larger microbial community (MBC and MBN) compared to the upslope clusters 2 and 3.

Results of the t-tests indicate that the BMUs are not necessarily good representatives of the typical (mean) microbial activity of each group (Table 3). Van Arkel and Kaleita [19] demonstrated that the BMUs identified through the K-means approach was a useful way to pinpoint individual locations to monitor for soil moisture. On the date of our soil sampling, however, selecting a single BMU from the cluster analysis appears to be less useful for identifying specific sampling locations for observing microbial activity. All of the BMUs had two or more soil microbial activity metrics that were statistically significantly different than the group mean. At the same time, microbial biomass carbon and microbial biomass nitrogen both showed significant agreement between BMU observation and group mean, suggesting that the BMU site was representative of the group behavior for these two metrics.

**Table 3:** At a significance level of p<0.05, whether or not there is a statistically significant difference between the mean of each group and that group’s BMU.

	Cluster 1	Cluster 2	Cluster 3
**Extractable dissolved organic carbon (ug C g^-1^ soil)**	No	Yes	No
**Microbial biomass carbon (ug C g^-1^ soil)**	No	No	No
**Percent total soil carbon**	Yes	Yes	No
**Microbial biomass N (ug N g^-1^ soil)**	No	No	No
**Percent total soil nitrogen**	Yes	Yes	Yes
**Specific carbon mineralization**	Yes	Yes	No
**Specific N mineralization**	No	Yes	Yes

## Conclusions

These data suggest that the sampling design for microbial monitoring should consider within-field landscape variability, and that the clustering approach is useful for doing so. While the single BMUs did not necessarily represent the microbial activity for their respective clusters, the soil analysis data showed statistically distinct microbial activity among clusters with different terrain and soils characteristics. The topography data used to generate the clusters can be obtained from high resolution LiDAR, increasingly available through regional government investment, or other surveys.

One limitation of this study is that we used soil and microbial samples from just one day, late in the season. Microbial communities in agricultural fields are known to be responsive to management activities, among other factors. For instance, Girvan et al. [38] followed the bacterial and fungal communities in a wheat field over the course of a year. They observed that between-sample microbial heterogeneity decreased under a mature crop but increased following harvesting and ploughing. Our soil samples were collected after harvest, and thus may reflect more heterogeneity than had we sampled at a different time. Furthermore, one might expect temporal or seasonal dynamics to affect the relationship between terrain attributes and microbial activity; this is an area for future work.

Overall, this research demonstrates the value of spatially-explicit sampling for the field of soil microbial ecology, which currently struggles to find meaningful and efficient sampling strategies.

## Supporting information

S1 File Soil, terrain, and microbial indicator data. This file includes easting and northing coordinates for each sample, the assigned cluster from the SOM, the moisture content (g/g), fresh weight to dry weight ratio, pH, MBC (ug C/ g DW), MBN (ug N/ g DW), Specific C mineralization, Specific N Mineralization, percent total carbon, percent total nitrogen, elevation (m), slope, aspect (degrees), plan curvature, horizontal-polarization EMI (mS/m), and vertical-polarization EMI (mS/m).
(XLSX)
